# Fallopian Tube Carcinosarcoma With Intrauterine Fluid Accumulation: MRI Findings, Diagnostic Challenges, and Long-Term Survival

**DOI:** 10.7759/cureus.87857

**Published:** 2025-07-13

**Authors:** Kaiji Inoue, Masahiro Koyama, Masanori Yasuda, Kosei Hasegawa, Eito Kozawa

**Affiliations:** 1 Department of Radiology, Saitama Medical University Hospital, Saitama, JPN; 2 Department of Pathology, Saitama Medical University International Medical Center, Saitama, JPN; 3 Department of Gynecologic Oncology, Saitama Medical University International Medical Center, Saitama, JPN

**Keywords:** dose-dense chemotherapy, fallopian tube carcinosarcoma, intrauterine fluid accumulation, long-term survival, mri

## Abstract

Fallopian tube carcinosarcomas are extremely rare biphasic malignancies with nonspecific clinical and imaging features, posing significant diagnostic challenges. We report a case of a heterologous carcinosarcoma originating from the fimbriated end of the fallopian tube, presenting as a right adnexal mass with intrauterine fluid accumulation in the absence of hydrosalpinx. Magnetic resonance imaging demonstrated heterogeneous high signal intensity on T2-weighted imaging, low signal intensity on T1-weighted imaging, and patchy enhancement on both early- and delayed-phase contrast-enhanced sequences. The tumor was surgically resected, and it was histologically diagnosed as a heterologous carcinosarcoma with chondrosarcomatous components. The patient underwent a two-stage surgery with dose-dense chemotherapy and achieved long-term survival, remaining recurrence-free for over 11 years. This case highlights the importance of considering fallopian tube carcinosarcoma in the differential diagnosis of adnexal tumors and demonstrates the potential favorable outcomes with timely intervention and appropriate treatment.

## Introduction

Carcinosarcomas are rare biphasic malignancies comprising both malignant epithelial and mesenchymal components. They most commonly arise in the uterine body, less frequently in the ovaries [1,2], and rarely in the fallopian tubes [3]. Fallopian tube carcinosarcomas, also known as malignant mixed Müllerian tumors, account for only 0.1%-0.5% of gynecologic malignancies [4] and primarily affect postmenopausal women.

Preoperative diagnosis of fallopian tube carcinosarcomas remains difficult because of their nonspecific clinical presentation and lack of distinct imaging features. The most common symptoms include abdominal pain and abnormal vaginal bleeding. These tumors are known for their more aggressive behavior, higher metastatic potential, and poorer prognosis compared to fallopian tube carcinomas. While magnetic resonance imaging (MRI) is essential for evaluating gynecologic malignancies, reports describing the MRI characteristics of fallopian tube carcinosarcomas are extremely limited in the English literature. Previously reported cases have described that these tumors exhibit a heterogeneous internal structure on T1- and T2-weighted images, heterogeneous enhancement on contrast-enhanced sequences, and high signal intensity on diffusion-weighted imaging. However, these features are shared with many other pelvic malignancies and are not specific to fallopian tube carcinosarcoma [4,5]. Although intrauterine fluid accumulation has been documented in fallopian tube carcinoma, its association with fallopian tube carcinosarcoma has not been well-established.

Herein, we present a rare case of fallopian tube carcinosarcoma with intrauterine fluid, focusing on its MRI features. This report aims to enhance understanding of this rare tumor and contribute to improving diagnosis accuracy and management strategies in future cases.

## Case presentation

Initial presentation and clinical evaluation

**Figure 1:**
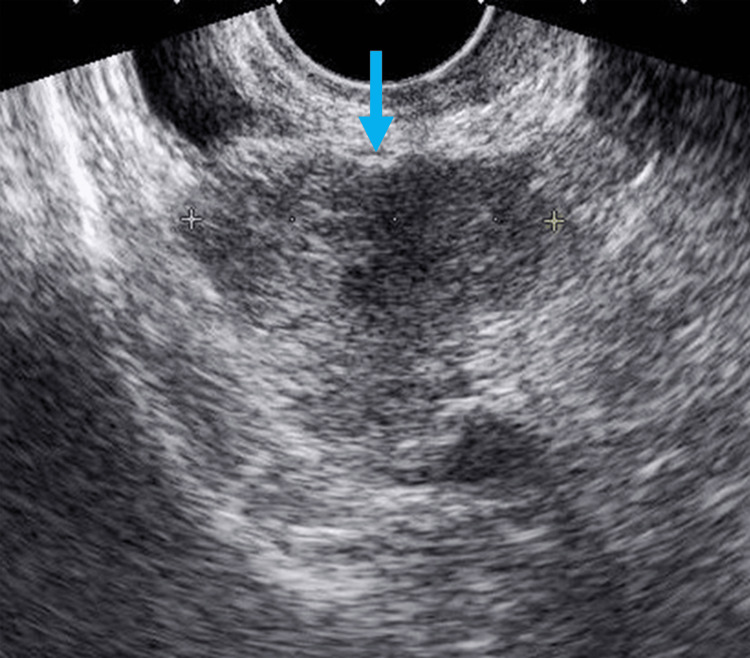
Ultrasonography findings Grayscale ultrasonography shows a hypoechoic mass on the right side of the pelvis (blue arrow).

A 68-year-old woman presented to our hospital with complaints of frequent urination persisting for over a month. Her physical examination showed no signs of abdominal pain, tenderness, or palpable masses. She experienced dysuria. Urinalysis revealed white blood cells at a 1+ level and a trace of occult blood (±), with no additional abnormalities. No vaginal discharge or bleeding was observed. Biochemical tests yielded normal results; however, a Papanicolaou (Pap) smear cytology revealed Class V atypia, indicative of carcinoma. Transvaginal pelvic ultrasonography identified a 40-mm solid hypoechoic mass on the right side of the uterine body, extending into the adnexal region with evidence of sound attenuation (Figure 1).

Serum tumor markers, including carcinoembryonic antigen (CEA), carbohydrate antigen 19-9 (CA 19-9), and cancer antigen 125 (CA-125), were within normal limits (Table 1). Given the possibility of ovarian malignancy, pelvic MRI for gynecological evaluation and whole-body computed tomography (CT) for staging were performed sequentially.

**Table 1:** Tumor marker levels CEA: carcinoembryonic antigen; CA 19-9: carbohydrate antigen 19-9; CA-125: cancer antigen 125

Tumor markers	Value	Reference range
CEA (ng/mL)	0.5	0.0-0.5
CA 19-9 (U/mL)	7	0.0-37
CA-125 (U/mL)	20.7	0.0-35

Imaging findings

**Figure 2:**
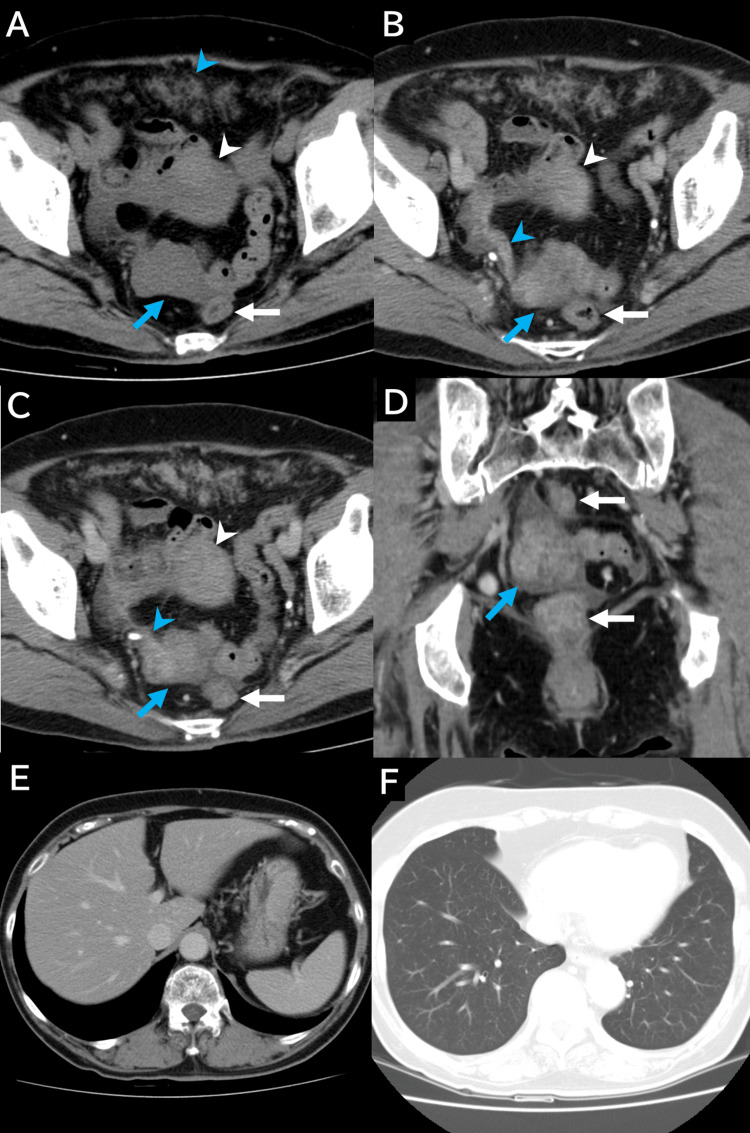
CT findings (A) On unenhanced axial CT, a 40 × 35 mm round mass (blue arrow) with homogeneous internal density equivalent to the uterus (white arrowhead) is observed on the right side of the rectum (white arrow). The mass is separated from the uterus (white arrowhead). Numerous ill-defined peritoneal nodules are present in the omentum (blue arrowhead). (B and C) On contrast-enhanced axial CT, the mass (blue arrow) located on the right side of the rectum (white arrow) shows mildly heterogeneous enhancement similar to that of the uterus (white arrowhead). The right ovarian vein (blue arrowhead) is continuous with the mass. (D) On contrast-enhanced coronal CT, the mass demonstrates a heterogeneous internal structure with patchy enhancement (blue arrow). The rectum is adjacent to the mass (white arrow). (E) On contrast-enhanced axial CT, no liver metastases or peritoneal nodules around the liver are observed. (F) No metastatic tumors are present in the lungs. CT: computed tomography

Unenhanced and contrast-enhanced CT imaging revealed a lobulated solid mass measuring approximately 40 × 35 mm on the right side of the rectum without calcification (Figure 2A). Contrast-enhanced CT demonstrated heterogeneous enhancement within the mass. The right ovarian vein, which ran continuously alongside the mass, was on the right side of the rectum (Figures 2B-2D). Multiple ill-defined disseminated nodules larger than 2 cm were observed in the omentum (Figure 2A). No liver or lung metastases were detected (Figures 2E, 2F).

**Figure 3:**
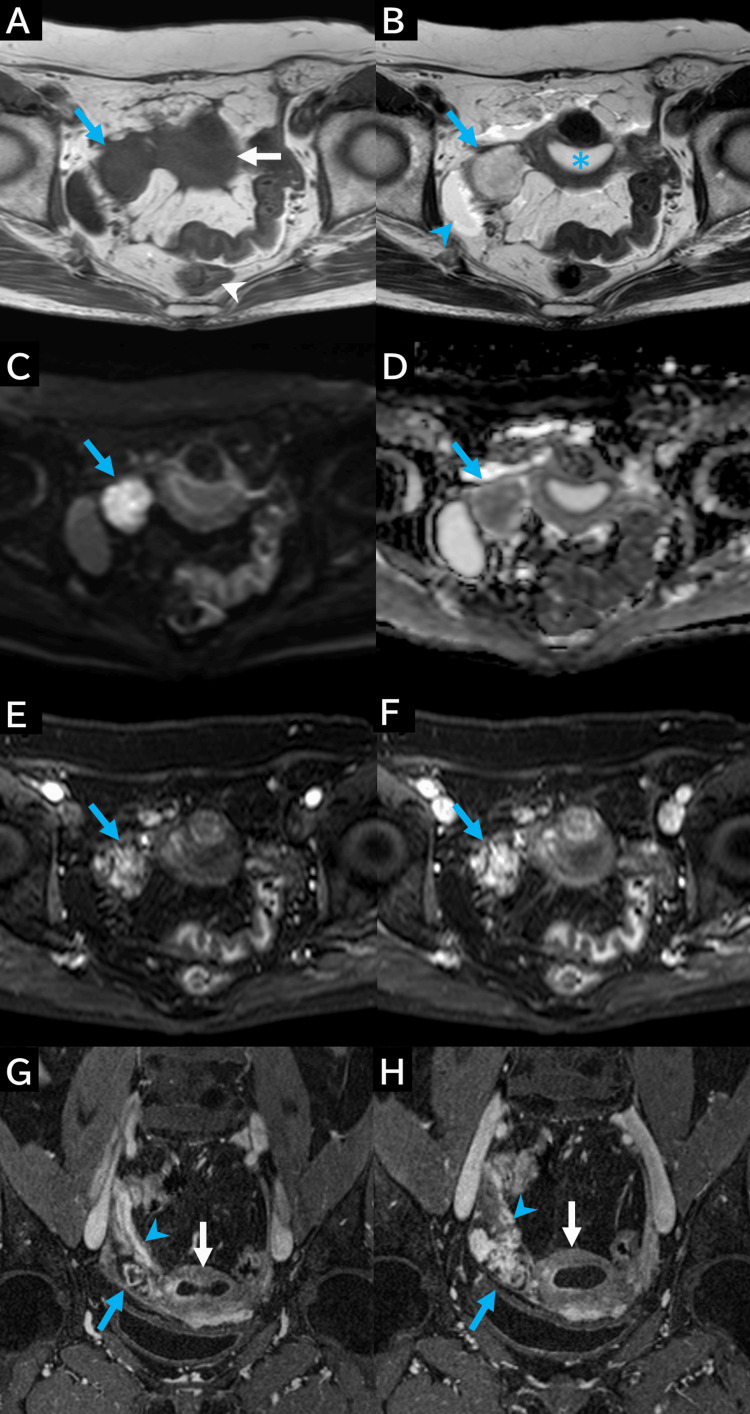
MRI findings (A) In contrast to CT findings, the mass is on the right side of the uterus (white arrow) and is separated from the rectum. On T1-weighted imaging, the mass (blue arrow) demonstrates signal intensity similar to that of the myometrium. (B) On axial T2-weighted imaging, the mass (blue arrow) shows heterogeneous high signal intensity with a low-signal-intensity rim, indicating a high-water content. Small amounts of fluid are observed around the mass (blue arrowhead) and within the endometrial cavity (blue asterisk). These findings are uncommon in postmenopausal women and suggest a fallopian tube lesion. (C) Axial diffusion-weighted image reveals a high-signal-intensity mass (blue arrow). (D) On the ADC map, the mass shows heterogeneous low signal intensity, suggesting a high likelihood of malignancy (blue arrow). (E) An early-phase (35 seconds) contrast-enhanced axial fat-saturated T1-weighted image reveals high signal intensity within the mass compared to the myometrium (blue arrow). (F) A delayed-phase (125 seconds) contrast-enhanced axial fat-saturated T1-weighted image reveals strong patchy high signal intensity within the mass (blue arrow). (G and H) A delayed-phase (180 seconds) coronal contrast-enhanced fat-saturated T1-weighted image reveals strong patchy high signal intensity within the mass (blue arrow). The right ovarian vein runs along the internal surface of the right adnexal tumor (blue arrowhead). The right ovarian vein (blue arrowhead) is continuous with the tumor on the right side of the uterus (white arrow). Although the tumor appears to be in a different location compared to that on the CT images, its continuity with the right ovarian vein and similar internal characteristics suggest that it is the same lesion. Fluid is present in the endometrial cavity (white arrow), which is uncommon in postmenopausal women and further supports the diagnosis of a fallopian tube lesion. ADC: apparent diffusion coefficient; MRI: magnetic resonance imaging

After CT imaging, T1-weighted MRI revealed that the mass had shifted from the right side of the rectum to the right side of the uterus, exhibiting iso-signal intensity relative to the myometrium (Figure 3A). T2-weighted imaging revealed heterogeneous high signal intensity compared to the uterine myometrium (Figure 3B). Fluid collection was observed in the uterine lumen; however, no fluid accumulation was detected in the fallopian tubes. Diffusion-weighted imaging (DWI) indicated high signal intensity (Figure 3C), and the apparent diffusion coefficient (ADC) map demonstrated heterogeneous low signal intensity (Figure 3D). Early and delayed-phase contrast-enhanced fat-saturated T1-weighted imaging identified patchy areas of high signal intensity within the mass, exhibiting a progressively increasing contrast pattern (Figures 3E, 3F). Delayed-phase coronal contrast-enhanced fat-saturated T1-weighted imaging illustrated that the right ovarian vein was adjacent to the mass at the right side of the uterus (Figure 3G, 3H).

Based on these imaging findings, there was an initial suspicion of epithelial ovarian carcinoma. However, the heterogeneous high signal intensity observed on T2-weighted imaging and the patchy high signal intensity seen on early- and delayed-phase contrast-enhanced T1-weighted imaging were consistent with uterine carcinosarcomas, suggesting a differential diagnosis of ovarian carcinosarcoma.

Initial surgery and pathological diagnosis

**Figure 4:**
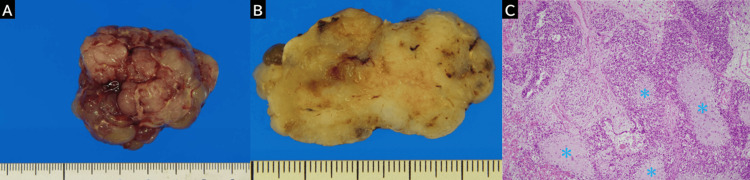
Pathological findings of heterologous-type fallopian tube carcinosarcoma (A) A surgical specimen from the right adnexectomy shows a 45 × 40 × 40 mm mass, with a bosselated exterior. (B) The cross-sectional view of the surgical specimen reveals a yellowish-white cut surface and a solid component. (C) Microscopic examination reveals islands of chondrosarcoma (asterisks) within areas of serous adenocarcinoma (hematoxylin and eosin, ×10). Complex papillae with striated epithelium and slit-like spaces are visible. Adenocarcinomatous components are intermixed with chondrosarcomatous elements, with cartilage comprising approximately 30% of each.

Given the clinical symptoms and imaging findings suggestive of a malignant tumor with omental dissemination, a right adnexectomy and partial omentectomy were performed. Peritoneal dissemination involving the perisigmoid and perihepatic regions, which had not been detected during preoperative imaging, was identified intraoperatively. The surgical specimen from the right adnexectomy comprised a 4 × 4 × 5 cm tumor mass, an ovary, and a fallopian tube. Intraoperative findings confirmed that the ovary was normal and that the mass was attached to the fimbriated end of the fallopian tube (Figure 4A). The cut surface of the mass appeared yellowish-white, smooth, and bosselated, with a solid component exhibiting minimal intratumoral necrosis and hemorrhage (Figure 4B). Histopathological analysis revealed islands of chondrosarcoma within areas of serous adenocarcinoma (Figure 4C). The cartilaginous elements constituted approximately 30% of each low-power field.

Immunohistochemical staining results were positive for MIB-1 and S100. Consequently, the tumor was diagnosed as a heterologous fallopian tube carcinosarcoma. According to the International Federation of Gynecology and Obstetrics (FIGO) 1988 staging system, which was in use at the time of surgery (2013), the tumor was classified as Stage IIIC because of the omental and perihepatic dissemination [6]. Although the tumor’s location differed slightly, the differential diagnoses from the imaging findings were consistent with the pathological diagnosis.

Postoperative chemotherapy and disease progression

Subsequently, the patient underwent four cycles of dose-dense chemotherapy consisting of weekly paclitaxel and tri-weekly carboplatin. This was because the peritoneal dissemination nodules in the sigmoid colon increased from 3 cm to 5 cm in size, as they were unresected during the first surgery. After completing the fourth cycle, CT revealed a reduction in the size of the peritoneal dissemination nodules to 3 cm and complete disappearance of the omental implants.

Interval debulking surgery and adjuvant therapy

Interval debulking surgery was performed, during which the uterus, left adnexa, and the portion of the sigmoid colon containing the peritoneal dissemination nodules were resected. Additionally, a 5-mm peritoneal dissemination nodule in the pouch of Douglas, not previously detected by CT, was identified and removed. No gross residual tumor remained at the conclusion of surgery, including in the perihepatic region. Cytological examination of the ascitic fluid collected during surgery revealed no malignant cells. Following the second surgery, the patient received three additional cycles of dose-dense chemotherapy with paclitaxel and carboplatin, as previously administered. During treatment, drug administration was postponed three times because of neutropenia. Other adverse effects, including constipation, diarrhea, abdominal pain, alopecia, nausea, and dysgeusia, were observed but remained within a tolerable range.

Follow-up and long-term outcome

After discharge, the patient underwent outpatient follow-up examinations every six months, including chest to pelvis CT and abdominal ultrasonography, to monitor for recurrence. Four years after discharge, routine follow-up revealed anemia. Further investigation with lower gastrointestinal endoscopy identified rectal cancer, for which the patient underwent endoscopic submucosal dissection. Since then, no recurrence has been observed, and the patient remained alive 11 years after the initial surgery.

## Discussion

Fallopian tube primary carcinosarcomas are extremely rare, accounting for only 0.1%-0.5% of all gynecologic malignancies and approximately 2.4% of all fallopian tube malignancies [7]. These tumors predominantly occur in postmenopausal women in their 50s to 60s, and rarely in those under 40 years of age [7]. The present case involved a 68-year-old woman, consistent with the typical age distribution. Clinical symptoms are often nonspecific, with common presentations including lower abdominal pain, abnormal vaginal bleeding, and abdominal distension, symptoms frequently reported in gynecologic diseases [8]. In the present case, the patient experienced only urinary frequency and dysuria, without abdominal pain or vaginal bleeding. Tumor marker levels were within the normal range, a finding not unusual in heterologous carcinosarcomas [1,7]. As reported in our case, approximately 50% of fallopian tube carcinosarcomas are diagnosed at FIGO stage III, based on the study by Xiao et al., in which 43 of 85 patients were diagnosed at this advanced stage [8]. Because of the aggressive nature of the disease, the average survival for stage III patients is markedly short, at only 16.1 months [8]. While no standard treatment is established, surgical resection and platinum-based chemotherapy have demonstrated efficacy in many cases [3].

Prognostic factors

Remarkably, the patient in this case survived for over 11 years since diagnosis. We believe several key factors contributed to this favorable outcome. First, interval debulking surgery achieved complete macroscopic tumor resection. According to Xiao et al., complete resection is essential for improving long-term survival of patients [8]. Second, the chemotherapy regimen of dose-dense weekly paclitaxel and tri-weekly carboplatin may have played a critical role [7-10]. While some studies report no significant difference between this and the conventional tri-weekly regimen [11], others suggest that Asian patients, while experiencing higher toxicity, achieve better treatment outcomes with the dose-dense regimen [12]. Taguchi et al. demonstrated that this approach is particularly effective in patients over 50 with stage II/III disease and residual tumors ≥1 cm, similar to our case [13]. Apart from neutropenia, the patient tolerated the treatment well, which likely enabled her to complete the full chemotherapy course. Third, close monitoring every six months, including imaging studies, facilitated the early detection and successful treatment of a subsequent rectal cancer. This comprehensive follow-up strategy may have improved the overall prognosis. Therefore, complete resection, appropriate chemotherapy, and intensive follow-up likely contributed to a survival period nearly eight times longer than the average for stage III disease.

Imaging findings and diagnostic challenges

Understanding the MRI characteristics of fallopian tube carcinosarcoma is crucial for early diagnosis. However, preoperative identification remains challenging due to overlapping imaging features with other malignancies of the ovary and fallopian tube and the rarity of reported cases [4,5,8,14]. In this case, the tumor exhibited low signal intensity on T1-weighted imaging, heterogeneous high signal intensity on T2-weighted imaging, and restricted diffusion on diffusion-weighted imaging. Contrast-enhanced MRI showed a patchy enhancement distinct from the myometrium, consistent with previous reports describing persistent enhancement in uterine carcinosarcomas during delayed phases [15-18]. Similar findings have been reported in fallopian tube carcinosarcomas, including heterogeneous internal enhancement and sustained contrast retention [4,5,8]. Typical uterine carcinosarcomas show low signal intensity on T1-weighted imaging, often with foci of high intensity due to hemorrhage, and heterogeneous moderate-to-high signal intensity on T2-weighted imaging, reflecting necrosis or hemorrhage [15,16]. In contrast, this case exhibited prominent T2 hyperintensity without significant necrosis or hemorrhage, suggesting the responsibility of other histologic components. Chondromatous and sarcomatous elements have been shown to contribute to high T2 signal intensity, even when chondroid tissue comprises less than 30% of the tumor [19]. In particular, non-mineralized chondrosarcoma elements exhibit a high water content characteristic of hyaline cartilage, leading to increased T2 signal [19]. Histopathological analysis in this case confirmed chondromatous components intermixed with adenocarcinomatous tissue, explaining the imaging features.

Clinical presentation and hydrops tubae profluens

In this case, the patient also presented with vaginal discharge, a symptom commonly seen in fallopian tube cancer. Intermittent discharge of clear fluid or blood, known as hydrops tubae profluens, results from the periodic accumulation and expulsion of fluid from a distended fallopian tube [3]. Intrauterine fluid accumulation and peritumoral ascites often accompany fallopian tube carcinomas because of tubal decompression [14]. Intrauterine fluid accumulation has also been reported in fallopian tube carcinosarcoma [4,5]. The presence of intrauterine fluid and peritumoral ascites in this case may indicate a similar pathophysiological mechanism. However, no clinical correlation was observed between discharge and the relief of colicky abdominal pain.

Differential diagnosis

Differentiating fallopian tube carcinosarcoma from fallopian tube carcinoma remains a diagnostic challenge. Fallopian tube carcinomas typically appear as sausage-like solid masses or mural nodules within a hydrosalpinx, often demonstrating homogeneous internal structures and peripheral contrast enhancement [14,20]. In contrast, the tumor in this case lacked marginal enhancement and exhibited marked heterogeneous high signal intensity on T2-weighted imaging, with sustained enhancement in the early and delayed phases. These findings serve as potential diagnostic clues; however, the overlap in imaging features between fallopian tube carcinosarcoma and other malignancies continues to hinder accurate preoperative diagnosis.

Limitations

Despite the detailed imaging and histopathological evaluation, this study has limitations. First, as a single case report, its findings cannot be generalized. Second, the extreme rarity of fallopian tube carcinosarcoma limits the availability of comparative imaging data. Lastly, the absence of genomic or molecular profiling hinders a deeper insight into the biological mechanisms underlying the favorable prognosis observed in this case. Accumulating further case reports with standardized imaging and histological descriptions is essential for establishing more reliable diagnostic criteria and treatment strategies.

## Conclusions

Fallopian tube carcinosarcoma is an extremely rare malignancy that presents diagnostic challenges due to nonspecific clinical and imaging features. In our case, intrauterine fluid accumulation, high T2 signal intensity of the lesion, and persistent contrast enhancement of the lesion in the early and delayed phases were observed. These imaging findings may offer important clues for early identification and differentiation from other pelvic tumors. However, definitive diagnosis requires surgical and histopathological confirmation. Additionally, the case also highlighted a favorable clinical outcome, with the patient surviving 11 years after treatment. Therefore, accumulating further case reports with standardized imaging descriptions is crucial for improving diagnostic accuracy and patient outcomes.
